# Does a change to an occupation with a lower physical workload reduce the risk of disability pension? A cohort study of employed men and women in Sweden

**DOI:** 10.5271/sjweh.4053

**Published:** 2022-10-29

**Authors:** Kathryn Badarin, Tomas Hemmingsson, Melody Almroth, Daniel Falkstedt, Lena Hillert, Katarina Kjellberg

**Affiliations:** 1 Unit of Occupational Medicine, Institute of Environmental Medicine, Karolinska Institutet, Stockholm, Sweden.; 2 Department of Public Health Sciences, Stockholm University, Stockholm, Sweden.; 3 Centre for Occupational and Environmental Medicine, Region Stockholm, Stockholm, Sweden

**Keywords:** ageing employee, disability benefit, exposure change, heavy manual job, heavy work, musculoskeletal, physical health, work ability, work condition

## Abstract

**Objective:**

This study aimed to examine if a change to an occupation with a lower physical workload reduces the risk of all-cause disability pension (DP) and musculoskeletal DP (MDP).

**Methods:**

The sample comprised 359 453 workers who were registered as living in Sweden in 2005 and aged 44–63 in 2010. Exposure to physical workload was measured from 2005–2010 by linking a mean value from a job exposure matrix to occupational codes. The mean values were then split into quartiles. All included participants had high exposure to physical workload (top quartile) from 2005–2007. A change in physical workload was measured as a change to (i) any lower quartile or (ii) medium-high or low quartiles from 2008–2010. DP cases were taken from register data from 2011–2016. Crude and multivariate Cox proportional-hazards regression models estimated sex-specific hazard ratios (HR) with 95% confidence intervals (CI).

**Results:**

Compared to workers with consistently high physical workload, a change to any lower quartile of physical workload was associated with a decreased risk of all-cause DP (men: HR 0.59, 95% CI 0.46–0.77, women: HR 0.63, 95% CI 0.52–0.76) and MDP (men: HR 0.52, 95% CI 0.31–0.89, women: HR 0.61, 95% CI 0.44–0.84). Older workers had the largest decreased risk for MDP. Generally, changing from high to low physical workload was associated with a greater reduced risk of DP than changing from high to medium-high physical workload.

**Conclusion:**

Changing to an occupation with lower exposure to physical workload was associated with reduced risks of DP and MDP among both sexes.

The length of working life is expanding in light of the ageing population. However, many workers report that they believe they will be unable to continue their current work tasks until retirement age (1). A Swedish study found that <50% of blue-collar workers and around 60–75% of white-collar workers reached expected retirement age for the Swedish population (65 years) (2). Blue-collar workers are disproportionately exposed to adverse work conditions, which could partly explain the disparity in exit rates compared to white-collar workers (2). Functional capacity generally declines by age and, therefore, the effects of adverse working conditions on workforce marginalization could be greater among older than younger workers (3). Workers aged ≥50 years in physically demanding jobs have reported that the imbalance between their physical capacity and job tasks is a factor pushing them out of working life (4). Strenuous physical work such as awkward postures, overall heavy physical workload or heavy lifting has been associated with musculoskeletal disorders (5) and exit from the labor market, often through disability pension (DP) (6–9).

Changing from a high to a lower level of exposure to physical workload could help reduce the risk of disability pension but this is, as yet, unknown. A prospective Swedish study found that male and female workers with a history of long-term sick leave who changed jobs had a higher likelihood of remaining in the labor market two to four years later, compared to workers who stayed in the same job (10). However, the study could not capture whether a change in occupation resulted in a change of exposure to workplace hazards.

Studies investigating the effects of a change in exposure to physical workload on labor market attachment are scarce. Two prospective studies based on data from the Helsinki Health Study (a sample of municipal employees, of which the majority were women) have explored effects of a self-reported change of exposure to physical workload using questionnaire data and physical health functioning (a fundamental factor for work ability) (11) or sickness absence (12). One study found that repeated or increased exposure to physical workload was associated with a greater risk of poorer physical health functioning, likewise, decreased physical workload was associated with reduced risk (11). The other study reported that decreased exposure to physical workload was associated with a reduced risk of sickness absence (12). A prospective study from The Netherlands reported that a favorable change in physical workload, measured through annual questionnaires, was associated with a reduced risk of exit from paid employment (three consecutive months out of paid employment) among workers with a chronic disease (13).

Only one previous study investigating the effects of changes in physical workload on DP has been found (14). This prospective Swedish study examined whether industry change among male construction workers (as a proxy for change in physical workload) – in the year they turned 45, 50 or 55 – was associated with a lower risk of DP at 60–64 years of age (14). The study classified the workers into heavy and less heavy occupations (based on cardiovascular load). A move away from the construction industry was associated with a lower risk of DP. The largest risk reductions were found among workers changing industry at 55 years. Unexpectedly, similar risk reductions were observed among workers in heavy and less heavy jobs. However, it is important to note the study did not have data on workers’ occupations and could only assume that industry change resulted in a lower exposure to physical workload.

To our knowledge, no previous studies have investigated if a change to lower physical workload reduces the risk of a DP among men and women in the total working population. Also, the effects of exposure to heavy physical workload on the musculoskeletal system could vary depending on sex/gender (15), thus the risks associated with exposure changes might also differ. It is, therefore, cogent to explore sex-specific risks associated with changes in physical workload on DP, which are, yet, unknown. Furthermore, in light of the evidence showing associations between heavy physical workload and poorer musculoskeletal health (5), changes in exposure to physical workload should also be investigated in relation to DP due to a musculoskeletal diagnosis.

This study aimed to examine whether a change to an occupation with a lower exposure to physical workload reduces the risk of a DP (all-cause and musculoskeletal) among middle-aged and older working men and women.

## Method

### Study population

This study used data from the Swedish Work, Illness, and labor-market Participation (SWIP) cohort. The SWIP cohort includes all individuals 16–64 years of age who were registered as living in Sweden in 2005, around 5.4 million people. The cohort was created through linkages of several registers. Sweden’s unique personal identity numbers, for all persons registered as living in Sweden, enables the data linkages. Statistics Sweden obtained and deidentified the data to protect confidentiality. Details of the SWIP cohort have previously been published (7). For this study, data from four registers were used. Information on birth, death, civil status, and migration were obtained from the Swedish total population register (16). The Longitudinal Integrated Database for Health Insurance and Labour Market Studies register (LISA) provided sociodemographic information (eg, occupation, educational attainment, birth country, and unemployment) for all 16–64-year-old persons living in Sweden from 1990 (17). However, occupational information was only available from 2005. The Micro Data for Analysis of the Social Insurance System (MIDAS) register provided information on compensations due to sick leave and DP (18). Finally, data on hospitalizations were taken from the Swedish national inpatient register (19).

### Participants and study design

**Figure 1:**
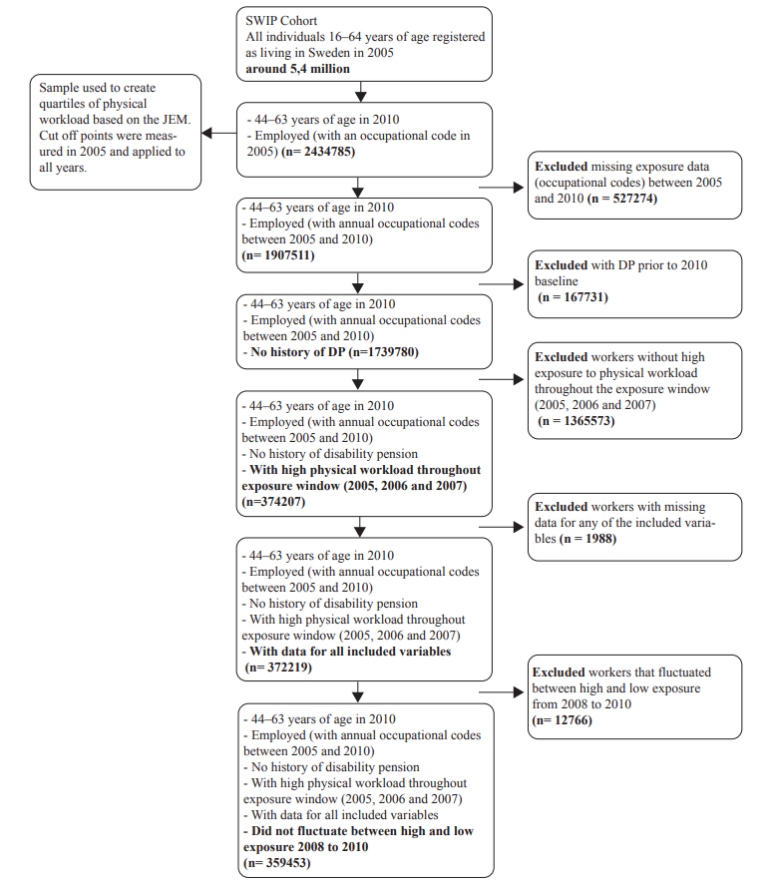
Sample selection

Workers born during 1947–1966 (44–63 years old in 2010) were selected for this study (N=2 434 785). This age group was chosen to try to capture those most at risk for a DP and those still eligible (<65 years) to claim a DP during the follow-up period of 2011–2016. A worker was defined as an individual with a Swedish Standard Classification of Occupation (SSYK) 96 code. SSYK codes are used to classify occupations (20) and were obtained for all study participants from the LISA register. For this study, the SSYK codes were used to estimate exposure to physical workload over a six-year period (2005–2010) using a job exposure matrix (JEM). The JEM provides a gender-specific aggregated measure of exposure to overall physical workload for 355 occupations. The JEM was constructed using the responses to eight questions included in the Swedish Work Environment Surveys 1997–2013. The questions relate to different aspects of physical work (heavy lifting (≥15 kg), physically strenuous work, fast breathing due to physical work, forward bent position, twisted position, working with hands above shoulder level, repetitive work and frequent bending and twisting) (21). Overall exposure to physical workload was estimated using an index score created by summing the scores of the responses to the eight questions on physical workload and calculating an overall mean value. The mean JEM values for overall physical workload were linked to the annual SSYK codes (occupations) for all participants from 2005 to 2010. To estimate level of exposure to physical workload over the six-year period, the mean JEM values for each year were split into sex-specific quartiles: high, medium-high, medium-low, and low. The cut-offs for the quartiles were based on the quartiles of physical load in 2005, and the same cut-off values were applied to the subsequent years. Workers missing an SSYK code for any of the years between 2005 and 2010 (N=527 274), with a DP prior to the end of 2010 (N=167 731), or missing data for any of included variables were excluded (N=1988) (figure 1). To explore the effect of a stable change from a high to a lower level of physical workload on DP, only workers exposed to a stable high level of heavy physical work – those in the highest quartiles of physical workload for a three-year period (2005–2007) (N=372 219) – were included in this study.

### Exposure: Change from a high to lower level of physical workload

**Figure 2:**
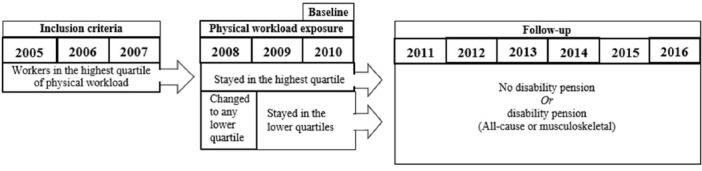
Study design

A change to a stable lower exposure to physical workload was measured as a change from the top quartile of physical workload to any of the lower quartiles in 2008 and remaining in the lower quartiles in 2009 and 2010 (figure 2).

It should be noted that workers could change to different occupations within the lower quartiles of physical workload between 2008 and 2010, but workers who fluctuated between high and lower physical workload were excluded (N=12 766). The final sample included (N=359 453) (figure 1).

A change from high to lower physical workload was further investigated by dividing the lower quartiles into two categories: medium-high physical workload and low physical workload (the low category included both the medium-low and low quartiles). In this analysis, workers who fluctuated between medium-high and low exposure between 2008 and 2010 were excluded (N=170).

### Outcome: Disability Pension

DP is a sickness compensation granted to 30–64-year-olds who, due to illness, injury, or disability, have a permanent impairment in work ability (22). To be eligible for DP, a medically certified reduction in work ability of ≥25% is required. DP can be granted in full or partially (three-quarter, one-half or one-quarter) depending on one’s work ability. In 2008, Sweden introduced more stringent eligibility requirements for DP, subsequently the number of granted applications reduced (2). In this study, DP cases were investigated between 2011 to 2016, ie, after the changes in eligibility requirements. Any first time, full or partial, DP during the follow-up period were included as a case. Workers with a DP prior to the end of the 2010 were excluded. Information on DP were obtained from the MiDAS register and two outcomes were explored: all-cause DP (any ICD 10 code) and DP due to a musculoskeletal diagnosis (ICD 10 codes M00–M99).

### Covariates

Several variables were taken from the LISA register for 2010. Educational attainment was divided into four groups: (i) primary and lower secondary school or less (≤9 years); (ii) secondary (10–11 years); (iii) upper-secondary (12 years); (iv) tertiary (≥13 years). Civil status was categorized as either married, unmarried, divorced, or widowed. Country of birth was dichotomized into born in or outside of Sweden. Unemployment five years before the start of follow-up (2006–2010) was divided into three groups: (i) 0 (ii) 1–365 and (iii) >365 days. Data on sick leave five years before the start of the follow-up were obtained from the MiDAS register, which provides data on sick leave episodes >14-days. Sick leave was divided into two groups i) 0 and ii) ≥1 episode. The in-patient register provided data on history of hospitalization for a psychiatric illness before the follow-up period, which was identified using ICD 10 codes F00–F99.

### Statistical analysis

First, we examined the distribution of the covariates across two exposure groups: (i) high physical workload and (ii) changed from high to any lower quartile of occupational physical workload. Second, the bivariate associations between the covariates and DP (all-cause or musculoskeletal diagnosis) were examined using Cox proportional-hazards regression, which produces hazard ratios (HR) with 95% confidence intervals (CI). Third, regression models were used to investigate the association between a change from a high to either a medium-high or low physical workload and DP. This analysis was conducted on the whole sample and stratified by age [middle-aged (44–53 years) and older (54–63 years)]. Person-time was calculated from 1 January 2011 until either emigration, old age pension, turning 65 years old, death, DP or the end of follow-up on 31 December 2016. Model 1 shows the crude results (adjusted for age). Model 2 is adjusted for age, education, civil status, country of birth, all-cause sick leave, unemployment, and hospitalization for a psychiatric illness. Fourth, we investigated the level of reduction of exposure to physical workload and the risk of DP. For this analysis the exposure had three groups: (i) high (reference category); (ii) medium-high, and (iii) low (a combination of workers in the medium-low or low quartiles). The last two groups were combined to increase the number of DP cases. Last, we examined the association between a change in physical workload and the risk of DP by including each confounding variable separately into the crude model (supplementary material 2). All analyses were stratified by sex and conducted using SAS 9.4 (SAS Institute, Cary, NC, USA).

## Results

The final sample included 359 453 workers. In total, 3.9% of men and 4% of women changed from high physical workload to any of the lower quartiles of physical workload. Table 1 shows the distribution of the covariates among workers who maintained high physical workload or changed to any of the lower quartiles of physical workload. Among both sexes, being younger, more highly educated and with an unemployment history was more common for workers who changed exposure than workers who maintained high exposure. The proportion of workers who were unmarried, born outside of Sweden, with a history of sick leave or a hospitalization for a psychiatric illness was similar in each exposure group.

**Table 1:** The distribution of the covariates across the exposure groups: (i) stable high physical workload (workers who remained in the highest quartile of physical workload (2005 to 2010) (ii) changed to lower PWL (a change from the topquartile to any of the lower quartiles in 2008 and remaining in the lower quartiles in 2009 and 2010).

	Men			Women		
	
	Change in exposure to physical workload			Change in exposure to physical workload		
	
	Stable high (N=177 399)	Changed high to lower (N=7116)	Total (N=184 515)	Stable high (N=167 881)	Changed high to lower (N=7057)	Total (N=174 938)
					
	N (%)	N (%)	N	N (%)	N (%)	N
Age (years)						
44–48	52 443 (30)	2501 (35)	54 944	47 587 (28)	2481 (35)	50 068
49–53	44 823 (25)	1921 (27)	46 744	43 995 (26)	1928 (27)	45 923
54–58	41 284 (23)	1525 (21)	42 809	40 269 (24)	1488 (21)	41 757
59–63	38 849 (22)	1169 (16)	40 018	36 030 (22)	1160 (16)	37 190
Country of birth						
Sweden	150 490 (85)	6033 (85)	156 523	134 656 (80)	5981 (85)	14 0637
Other	26 909 (15)	1083 (15)	27 992	33 225 (20)	1076 (15)	34 301
Educational level ^a^						
Tertiary	10 750 (6)	746 (11)	11 496	13 820 (8)	1289 (18)	15 109
Upper-secondary	18 467 (10)	901 (13)	19 368	25 902 (15)	1231 (17)	27 133
Secondary	91 818 (52)	3569 (50)	95 387	90 428 (54)	3531 (50)	93 959
Primary	56 364 (32)	1900 (27)	58 264	37 731 (23)	1006 (14)	38 737
Civil status						
Married	87 189 (49)	3715 (52)	90 904	90 877 (54)	3618 (51)	94 495
Unmarried	60 604 (34)	2173 (31)	62 777	39 290 (23)	1754 (25)	41 044
Divorced	28 154 (16)	1167 (16)	29 321	32 919 (20)	1527 (22)	34 446
Widowed	1452 (1)	61 (1)	1513	4795 (3)	158 (2)	4953
Hospitalisation for a psychiatric illness ^b^						
No	168 823 (95)	6780 (95)	175 603	160 462 (96)	6705 (95)	167 167
Yes	8576 (5)	336 (5)	8912	7419 (4)	352 (5)	7771
Unemployment (days) ^b^						
0	139 347 (79)	4949 (70)	144 296	140 729 (84)	4971 (70)	145 700
1–365	24 591 (14)	1406 (20)	25 997	18 859 (11)	1502 (21)	20 361
>365	13 461 (8)	761 (11)	14 222	8293 (5)	584 (8)	8877
Sick leave (all-cause) episodes ^b^						
0	159 806 (90)	6487 (91)	166 293	151 869 (91)	6513 (92)	158 382
≥1	17 593 (9)	629 (9)	18 222	16 012 (10)	544 (8)	16 556

a Primary = ≤9 years; secondary = 10–11 years; upper-secondary = 12 years; tertiary = ≥13years.

b Prior to start of follow-up.

Being older was associated with increased risks of DP, more notably for musculoskeletal DP (table 2). Being born outside of Sweden, having lower educational attainment, hospitalization for a psychiatric illness, unemployment, or sick leave were also associated with increased risks of DP for both sexes. Being unmarried was associated with increased risks of DP for men and being divorced was associated with increased risks for both sexes.

**Table 2:** Association between confounders and disability pension (DP). All models were adjusted for age.

	DP (all-cause DP)		DP (musculoskeletal)	
	
	Men (N=184 515)	Women (N=174 938)	Men (N=184 515)	Women (N=174 938)
			
	HR (95% CI)	HR (95% CI)	HR (95% CI)	HR (95% CI)
Age (years)				
44–48	1	1	1	1
49–53	1.56 (1.44–1.70)	1.21 (1.14–1.29)	1.64 (1.37–1.96)	1.35 (1.21–1.50)
54–58	2.49 (2.30–2.70)	1.46 (1.39–1.55)	3.13 (2.65–3.70)	2.08 (1.85–2.29)
59–63	2.86 (2.60–3.15)	1.45 (1.33–1.57)	17.29 (14.12–21.18)	11.42 (9.91–13.15)
Country of birth				
Sweden	1	1	1	1
Other	1.92 (1.80–2.05)	1.73 (1.64–1.82)	2.21 (1.94–2.51)	1.78 (1.64–1.93)
Educational Level ^a^				
Tertiary	1	1	1	1
Upper-secondary	0.96 (0.83–1.12)	1.28 (1.15–1.43)	1.09 (0.80– 1.50)	1.57 (1.29–1.92)
Secondary	0.99 (0.87–1.11)	1.33 (1.20–1.46)	1.16 (0.88–1.51)	1.68 (1.41–2.01)
Primary	1.21 (1.07–1.37)	1.84 (1.66–2.04)	1.58 (1.20–2.06)	2.55 (2.12-3.07)
Civil status				
Married	1	1	1	1
Unmarried	1.40 (1.31–1.49)	0.98 (0.93–1.04)	0.84 (0.73–0.97)	0.71 (0.64–0.79)
Divorced	1.68 (1.56–1.82)	1.46 (1.38–1.54)	1.28 (1.10–1.49)	1.09 (0.99–1.20)
Widowed	1.34 (0.99–1.82)	1.15 (0.99–1.33)	1.12 (0.62–2.03)	0.79 (0.61–1.02)
Hospitalisation for a psychiatric illness ^b^				
No	1	1	1	1
Yes	5.07 (4.70–5.46)	4.34 (4.10–4.66)	2.77 (2.29–3.35)	1.93 (1.67–2.23)
Unemployment (days) ^b^				
0	1	1	1	1
1–365	2.46 (2.30–2.63)	2.98 (2.83–3.15)	2.91 (2.55–3.32)	3.04 (2.77–3.33)
>365	2.49 (2.29–2.71)	2.57 (2.37–2.78)	2.36 (1.97–2.83)	2.63 (2.29–3.02)
Sick leave episodes ^b^				
0	1	1	1	1
≥1 episode	1.36 (1.25–1.48)	1.17 (1.08–1.26)	1.28 (1.13–1.56)	1.08 (1.01–1.19)

a Primary = ≤9 years; secondary = 10–11 years; upper–secondary = 12 years; tertiary = ≥13years.

b Prior to start of follow-up.

During the follow-up, 4756 cases of all-cause DP were observed among men and 7103 among women. Of these cases, 1124 were granted due to musculoskeletal diagnoses for men and 2483 for women.

Compared to workers with high physical workload, a change from high to lower physical workload (any of the lower quartiles) was associated with a decreased risk of all-cause DP (men: HR 0.64 95% CI 0.53–0.76, women: HR 0.71, 95% CI 0.62–0.82) and musculoskeletal DP (men: HR 0.61, 95% CI 0.42–0.89,women: HR 0.66, 95% CI 0.52–0.84) (table 3, model 1). For both sexes, the estimates for DP (all-cause and musculoskeletal diagnosis) decreased further after adjusting for the aforementioned confounders (table 3, model 2). The crude and adjusted age-stratified analyses showed that middle-aged men who changed to lower physical workload had a larger reduction in risk of all-cause DP than the older workers. Contrarily, older men that changed to lower physical workload had a markedly lower risk of musculoskeletal DP than workers who changed exposure in middle-age. Among women, there were no clear differences in the risks of DP between the age groups.

**Table 3:** The risk of disability pension (DP) among workers who changed from high to low physical workload (PWL) (any combination of lower quartile) compared to workers with stable high physical workload. [HR=hazard ratio; CI=confidence interval]

Age	PWL	DP (all-cause)				DP (musculoskeletal)			
	
		Cases		Model 1 ^a^	Model 2 ^b^	Cases		Model 1 ^a^	Model 2 ^b^
					
		N	%	HR (95%CI)	HR (95%CI)	N	%	HR (95%CI)	HR (95%CI)
Men (N=184 515)									
All	High	4638	2.6	1	1	1097	0.6	1	1
	Changed to low	118	1.7	0.64 (0.53–0.76)	0.59 (0.46–0.77)	27	0.4	0.61 (0.42–0.89)	0.52 (0.31–0.89)
Middle-aged ^c^	High	2181	2.2	1	1	475	0.5	1	1
	Changed to low	52	1.2	0.53 (0.40–0.69)	0.52 (0.39–0.68)	17	0.4	0.79 (0.49–1.29)	0.74 (0.46–1.21)
Older ^d^	High	2457	3.0	1	1	622	0.8	1	1
	Changed to low	66	2.4	0.76 (0.59–0.97)	0.63 (0.43–0.92)	10	0.4	0.44 (0.24–0.82)	0.21 (0.07–0.66)
Women (N=174 938)									
All	High	6893	4.1	1	1	2415	1.4	1	1
	Changed to low	210	3.0	0.71 (0.62–0.82)	0.63 (0.52–0.76)	68	1.0	0.66 (0.52–0.84)	0.61 (0.44–0.84)
Middle-aged ^c^	High	3902	4.3	1	1	1275	1.4	1	1
	Changed to low	124	2.8	0.66 (0.55–0.79)	0.61 (0.51–0.73)	41	1.0	0.68 (0.50–0.92)	0.64 (0.47–0.87)
Older ^d^	High	2991	3.9	1	1	1140	1.5	1	1
	Changed to low	86	3.2	0.80 (0.64–0.99)	0.58 (0.42–0.79)	27	1.0	0.65 (0.44–0.95)	0.56 (0.33–0.93)

a Adjusted for age.

b Adjusted for age, education, civil status, country of birth, sick leave* (all–cause), unemployment*, hospitalisation for a psychiatric illness* *=Prior to start of follow-up.

c 44–53 years.

d 54–63 years.

When investigating the effects of the addition of each confounder separately on the crude estimates, controlling for the confounding of civil status and unemployment strengthened the estimated effect of changing from heavy to lower physical workload occupations; unemployment contributed most to this. Adjusting for country of birth or education slightly increased the crude estimate, but only among women, and hospitalization for a psychiatric illness or sick leave had little effect on the crude estimate (supplementary material table S1).

Compared to workers with continued high physical workload, a change to medium-high physical workload was associated with a similar reduced risk of all-cause DP among men (HR 0.80, 95% CI 0.65–1.00) and women (HR 0.81, 95% CI 0.70–0.95) (table 4). A change from high to low physical workload (medium-low and low quartiles combined) was associated with a larger reduced risk of all-cause DP than a change to medium-high among both sexes (men; HR 0.44, 95% CI 0.31–0.61 and women, HR 0.49, 95% CI 0.36–0.68). After adjustment (model 2), the HR for all-cause DP for those who changed from high to medium-high physical workload reduced further and those who changed from high to low physical workload increased.

**Table 4:** The risk of disability pension (DP) among workers who changed from a high to stable low physical workload compared to workers with stable high physical workload. Sample only includes workers with stable high exposure or changed to stable medium-low or low exposure (N=359 283). [HR=hazard ratio; CI=confidence interval]

	DP (all-cause DP)				DP (musculoskeletal)			
	
	Cases		Model 1 ^a^	Model 2 ^a^	Cases		Model 1 ^a^	Model 2 ^b^
					
	N	%	HR (95% CI)	HR (95% CI)	N	%	HR (95% CI)	HR (95% CI)
Men (N=184 439)								
Stable high	4638	2.6	1	1	1097	0.6	1	1
High to medium-high	83	2.1	0.80 (0.65–1.00)	0.68 (0.55–0.85)	17	0.4	0.68 (0.42–1.10)	0.56 (0.35–0.91)
High to low ¦	35	1.1	0.44 (0.31–0.61)	0.50 (0.36–0.69)	10	0.3	0.52 (0.28–0.97)	0.58 (0.31–1.08)
Women (N=174 844)								
Stable high	6893	4.1	1	1	2415	1.4	1	1
High to medium-high	169	3.4	0.81 (0.70–0.95)	0.71 (0.61–0.82)	58	1.2	0.80 (0.62–1.04)	0.73 (0.56–0.94)
High to low ¦	41	2.1	0.49 (0.36–0.68)	0.53 (0.38–0.70)	10	0.5	0.35 (0.19–0.65)	0.37 (0.20–0.68)

a Adjusted for age.

b Adjusted for age, education, civil status, country of birth, sick leave* (all–cause), unemployment*, hospitalisation for a psychiatric illness*. *=Prior to start of follow-up.

§ Low group includes workers in both the medium-low and low quartiles of physical workload.

The reduced risk of musculoskeletal DP was greater among workers who changed from a high to low physical workload (men; HR 0.52, 95% CI 0.28–0.97 and women, HR 0.35, 95% CI 0.19–0.65) than those who changed from high to medium-high physical workload (men; HR 0.80, 95% CI 0.62–1.04 and women, HR 0.68, 95% CI 0.42–1.10), compared to workers with consistently high physical workload (table 4). After adjustment, this pattern remained for women, but not for men.

## Discussion

### Summary

This study found that a change from a high to a lower level of physical workload (any of the lower quartiles) was associated with a reduced risk of DP (all-causes and musculoskeletal diagnoses) among men and women. Middle-aged men who changed to lower physical workload had a slightly larger reduction in risk of all-cause DP than the older workers. Conversely, older men who changed to lower physical workload had a markedly lower risk of musculoskeletal DP than workers who changed exposure in middle-age. Among women, there were no clear differences in the risks of DP between the age groups. We also found that a change from high to either medium-high or low physical workload (medium-low and low quartiles combined) was associated with a reduced risk of DP (all-causes and musculoskeletal diagnoses). In general, a change from high to low physical workload was associated with the greatest reduced risk of all-cause DP and DP with a musculoskeletal diagnosis.

### Comparison with previous studies

To our knowledge, this is the first prospective study on a total working population to find that a change from a high to a lower exposure of physical workload was associated with a reduced risk of DP (all-cause or musculoskeletal). Our findings are in line with an existing prospective Swedish study by Söderberg et al (14) who found that a move away from heavy or less heavy construction jobs was associated with a lower risk of DP. Söderberg et al also showed that the largest reductions in the relative risks for DP were found among older workers (55 years). Their findings are in accordance with the results of this present study for the relationship between a reduction in physical workload and DP with a musculoskeletal diagnosis among older men. However, we found that men aged <55 years who changed to lower physical workload had a lower risk of all-cause DP than older male workers. Further, Söderberg et al (14) could only assume that an industry change resulted in a change in physical workload and did not explore diagnosis-specific DP or include female workers. The findings of this present study build upon the existing knowledge by using a somewhat more precise measure of a change in physical workload and exploring diagnosis-specific DP among men and women in the general working population.

The decreased risk of DP among workers who changed to lower physical workload found in this study is also in line with the results from two existing prospective studies on changes in physical workload and labor market marginalization. A Finnish study found that a reduction in physical workload was associated with a decreased risk of sickness absence among municipal employees (12). A study from The Netherlands reported that a favorable change in physical workload was associated with a reduced risk of exit from paid employment among workers with a chronic disease, mostly exit via unemployment or early retirement (13).

In this study, a change in physical workload was measured through a change in JEM score linked to an occupational code. Thus, a change in exposure was captured through a change in occupation. Söderberg et al (14) theorized that several factors (eg, health, higher qualifications, or personality type) could obscure the association between a change away from the construction industry and DP. If a large proportion of the workers who changed from an occupation with high exposure to physical workload to one with low exposure had poor health this could underestimate the reduced risk of DP associated with a reduction in physical workload. The opposite could be found, if more workers with good health changed to occupations with lower physical workload. However, in this study, adjusting for the potential confounding by poorer health measured by hospitalization for a psychiatric illness or sick leave had little effect on the crude estimate. However, musculoskeletal-related disorders are often treated by general practitioners, and we did not have access to outpatient data in our register-based cohort.

### Strengths and weaknesses

A strength of this study is the large study population that enabled the selection of a sample of workers with an accumulation of exposure to heavy physical workload at the occupational level. Furthermore, register-based studies, such as this one, do not suffer from attrition bias. The use of the JEM is another strength of this study. The JEM allowed us to estimate exposure to physical workload using annual data over a six-year period and examine the relationship between the level of reduction in physical workload and risk of DP. The JEM also helped eliminate self-report bias, as the data used to create the JEM are taken from a different sample than the one under investigation. However, the JEM is constructed on self-reported data, which is typically perceived as less accurate than more objective methods eg, accelerometry (24).

The JEM provides an aggregated measure of physical workload at an occupational level. Thus, the variation of physical workload between workers within an occupation is not captured. When investigating changes in exposure to physical workload using the JEM, as in this study, any changes through changing work tasks within the same job or changing employment but keeping the same occupational title (which could change the organization of one’s work) cannot be captured. It is also possible that some workers changed to less heavy working conditions but did not change occupation. These workers could have been misclassified as having stable high exposure, thus, our findings could be an underestimation of the true risks. A further weakness is potential residual and uncontrolled confounding as we are unable to account for many lifestyle factors eg, body mass index, smoking or leisure-time physical activity that could confound the relationship between change in physical workload and DP. Adjusting for education, however, could be viewed as a crude proxy for lifestyle factors, as such factors differ between socioeconomic groups in Sweden (25).

Our strict inclusion criterion was chosen to try capture workers who actually changed from high to lower physical workload. As a result, the number of workers who changed physical workload was small. Moreover, a large majority of the working population were excluded, which may limit the generalizability of our finding to those with the highest exposure to physical workload. Future studies could consider investigating changes in exposure among workers with medium high physical workload, which has also been associated with an increased risk of DP (7), or effects of exposure changes in any direction.

It should also be noted that it was difficult to pinpoint the optimal time point for the included covariates to be measured. Our inclusion criteria were between 2005 to 2007 and a stable change in exposure was measured in 2008 to 2010. Measuring the confounders in the last year before the follow-up period or as broad categories over a five-year period (2006 to 2010) gave a general indication for each confounder.

### Interpretation of results

Strenuous physical work has been associated with poorer musculoskeletal health (5) and exit from the labor market, often through DP (6–9). This study showed that a change to an occupation with lower physical workload was associated with a reduced risk of DP. We also found that the largest changes in physical workload were associated with the largest risk reductions. This could mean that a reduction in physical workload creates a better balance between workers’ health, functional capacity and work demands. This, in turn, could improve long-term work ability, and, in this way, prevent involuntary exits from work through DP.

Several factors could stand behind a change to an occupation with lower physical workload. We found that workers with higher education changed to jobs with lower physical workload more often than workers with lower education, which is in line with previous literature (23). However, adjusting for education only slightly changed the crude estimate, and only among women. Other factors include poor health and vocational rehabilitation strategies, as workers may not be able to return to their previous job. However, the proportion of workers with a history of sick leave was similar among workers who changed occupation and those who did not. Previous unemployment, however, was more prevalent among workers who changed exposure and had the largest effect on the association between reduced physical workload and DP.

When we looked into the type of occupations workers changed to, we found that management-level jobs made up many of the top ten jobs in the lowest quartiles of physical workload (supplementary table S2). Thus, career development could be a factor that drives a change to an occupation with lower exposure to physical workload. This could partly explain the strong reduction in risk for DP as workers with the possibility to advance to managerial positions could be a selected group of workers with advantageous health and lifestyle factors. However, as mentioned above, we did not find an attenuated effect estimate after adjusting for our chosen confounders, which indicates that the change to lower exposure to physical workload played a beneficial role in reducing the risk of DP. That said, residual and uncontrolled confounding should always be considered when interpreting the results of cohort studies.

Lowering exposure may be a more practical approach than trying to eliminate it. Our findings indicate that a smaller reduction in exposure to physical workload (from high to medium-high exposure) was associated with a reduced risk of DP. This finding is important to inform strategies that aim to maintain the health and prolong the working life of workers who are known to have a high risk of DP.

We found that older workers, particularly older men, who reduced their physical workload had the largest risk reduction for musculoskeletal DP. Older workers are more prone to musculoskeletal disorders than younger workers. This increased vulnerability is largely due to an increased imbalance between a worker’s job demands and their physical capacity (3). Therefore, it is comprehensible that older workers would have the greatest musculoskeletal health-related benefit from reducing their physical workload, which supports the findings of a previous study (14).

### Concluding remarks

Changing to an occupation with lower exposure to heavy physical workload was associated with a reduced risk of DP (all-cause and musculoskeletal) among both sexes. Older workers seemed to have the largest gain from reducing exposure to physical workload regarding musculoskeletal DP. Additionally, we found that a larger reduction in physical workload was associated with a greater reduced risk of all-cause DP and DP with a musculoskeletal diagnosis than a smaller reduction.

## Supplementary material

Supplementary material

## References

[1] EU-OSHA (2019). Work-related musculoskeletal disorders:prevalence, costs and demographics in the EU National report:Sweden.

[2] Kadefors R, Nilsson K, Rylander L, Г–Stergren P-O, Albin M (2018). Occupation, gender and work-life exits:a Swedish population study. Ageing Soc.

[3] Okunribido O, Wynn T, Lewis D (2010). Is age/ageing a risk factor for work-related musculoskeletal disorders?A literature review. Cont Ergo and Human Factors.

[4] Andersen LL, Jensen PH, Sundstrup E (2020). Barriers and opportunities for prolonging working life across different occupational groups:the SeniorWorkingLife study. Eur J Public Health.

[5] da Costa BR, Vieira ER (2010). Risk factors for work-related musculoskeletal disorders:A systematic review of recent longitudinal studies. Am J Ind Med.

[6] Ervasti J, Pietiläinen O, Rahkonen O, Lahelma E, Kouvonen A, Lallukka T (2019). Long-term exposure to heavy physical work, disability pension due to musculoskeletal disorders and all-cause mortality:20-year follow-up-introducing Helsinki Health Study job exposure matrix. Int Arch Occup Environ Health.

[7] Falkstedt D, Hemmingsson T, Albin M, Bodin T, Ahlbom A, Selander J (2021). Disability pensions related to heavy physical workload:a cohort study of middle-aged and older workers in Sweden. Int Arch Occup Environ Health.

[8] Kjellberg K, Lundin A, Falkstedt D, Allebeck P, Hemmingsson T (2016). Long-term physical workload in middle age and disability pension in men and women:a follow-up study of Swedish cohorts. Int Arch Occup Environ Health.

[9] Lahelma E, Laaksonen M, Lallukka T, Martikainen P, Pietiläinen O, Saastamoinen P (2012). Working conditions as risk factors for disability retirement:a longitudinal register linkage study. BMC Public Health.

[10] Nordström K, Ekberg K, Hemmingsson T, Johansson G (2014). Sick leave and the impact of job-to-job mobility on the likelihood of remaining on the labor market--a longitudinal Swedish register study. BMC Public Health.

[11] Mänty M, Kouvonen A, Lallukka T, Lahti J, Lahelma E, Rahkonen O (2015). Changes in working conditions and physical health functioning among midlife and ageing employees. Scand J Work Environ Health.

[12] Saastamoinen P, Laaksonen M, Lahelma E, Lallukka T, Pietiläinen O, Rahkonen O (2014). Changes in working conditions and subsequent sickness absence. Scand J Work Environ Health.

[13] Schram JL, Robroek SJ, Ots P, Brouwer S, Burdorf A, van Zon SK (2020). Influence of changing working conditions on exit from paid employment among workers with a chronic disease. Occup Environ Med.

[14] Söderberg M, Stattin M, Robroek SJ, Burdorf A, Järvholm B (2021). Industry mobility and disability benefits in heavy manual jobs:A cohort study of Swedish construction workers. Scand J Work Environ Health.

[15] Côté JN (2012). A critical review on physical factors and functional characteristics that may explain a sex/gender difference in work-related neck/shoulder disorders. Ergonomics.

[16] Ludvigsson JF, Almqvist C, Bonamy AK, Ljung R, Michaëlsson K, Neovius M (2016). Registers of the Swedish total population and their use in medical research. Eur J Epidemiol.

[17] Ludvigsson JF, Svedberg P, Olén O, Bruze G, Neovius M (2019). The longitudinal integrated database for health insurance and labor market studies (LISA) and its use in medical research. Eur J Epidemiol.

[18] The Swedish Social Insurance Agency F (2021). MiDAS.

[19] Socialstyrelsen The National Patient Register Socialstyrelsen;(2019).

[20] SCB SS ([2021]). SSYK 1996 Standard for svensk yrkesklassificering 1996 (Swedish Standard Classification of Occupations 1996). SCB statistics Sweden;2001.

[21] Badarin K, Hemmingsson T, Hillert L, Kjellberg K (2021). Physical workload and increased frequency of musculoskeletal pain:a cohort study of employed men and women with baseline occasional pain. Occup Environ Med.

[22] Forsakringskassan.se (2021). Sickness Compensation The Swedish Social Insurance Agency.

[23] Nordic Council of Ministers NC-o (2010). Labour Market Mobility in Nordic Welfare States.

[24] Wells R, Norman R, Neumann P, Andrews D, Frank J, Shannon H (1997). Assessment of physical work load in epidemiologic studies:common measurement metrics for exposure assessment. Ergonomics.

[25] Mäki NE, Martikainen PT, Eikemo T, Menvielle G, Lundberg O, Östergren O (2014). EURO-GBD-SE consortium members. The potential for reducing differences in life expectancy between educational groups in five European countries:the effects of obesity, physical inactivity and smoking. J Epidemiol Community Health.

