# Supplementary material for: Does a change to an occupation with a lower physical workload reduce the risk of disability pension? A cohort study of employed men and women in Sweden
Source: Scand J Work Environ Health. 2022 Oct 29;48(8):662–71. doi: 10.5271/sjweh.4053 (PMC10546609; doi:10.5271/sjweh.4053)
Supplement: Supplementary material [file SJWEH-48-662-S001.pdf]

Does a change to an occupation with a lower physical workload reduce the risk of disability pension? A cohort study of employed men and women in Sweden[1]

by Kathryn Badarin, MSc,[2] Tomas Hemmingsson, PhD, Melody Almroth, PhD, Daniel Falkstedt, PhD, Lena Hillert, MD, Katarina Kjellberg, PhD

1. *Supplementary Material*
2. *Correspondence to: Kathryn Badarin MSc, Unit of Occupational Medicine, The Institute of Environmental Medicine (IMM), Karolinska Universitet, Stockholm, Sweden. [E-mail: kathryn.badarin@ki.se]*

Table S1. The association between a change in exposure physical workload and all-cause disability pension when adjusting for each confounding variable

			Crude*	Country of birth	Completed Education	Civil status	Hospitalisation for a psychiatric illness	Unemployment	Sick leave
	Physical workload		HR (95%CI)	HR (95%CI)	HR (95% CI)	HR (95% CI)	HR (95% CI)	HR (95%CI)	HR (95% CI)
**Men (n=184515)**	Stable high	4638/177399	1	1	1	1	1	1	1
	high to low	118/7116	0.64 (0.53–0.76)	0.64 (0.53–0.76)	0.64 (0.53–0.77)	0.60 (0.41–0.88)	0.64 (0.53–0.77)	0.58 (0.48–0.69)	0.64 (0.53–0.76)
**Women (n=174938)**	Stable high	6893/167881	1	1	1	1	1	1	1
	high to low	210/7057	0.71 (0.62–0.82)	0.73 (0.64–0.84)	0.75 (0.65–0.86)	0.66 (0.52–0.84)	0.70 (0.61–0.80)	0.59 (0.52–0.68)	0.71(0.62–0.81)

*All analysis adjusted for age

Table S2. Ten most common occupations in the quartiles of physical workload in 2008

High	Men	Women
1	Carpenters and joiners	Assistant nurses and hospital ward assistants
2	Stock clerks and storekeepers	Helpers and cleaners in offices, hotels and other establishments
3	Motor vehicle mechanics and fitters	Helpers in restaurants
4	Other machine operators and assemblers	Shop sales person, food stores
5	Other sale and services elementary occupations	Cooks
6	Upholsters and related workers	Manufacturing labourers
7	Rail and road construction workers	Other sales and services elementary occupations

8	Welders and flame cutters	Stock clerks and storekeepers
9	Mail carriers and sorting clerks	Mail carriers and sorting clerks
10	Manufacturing labourers	Other machine operators and assemblers
Med-high	Men	Women
1	Building caretakers	Home based personal care and related workers
2	Heavy truck and lorry drivers	Attendants, psychiatric care
3	Agricultural or industrial machinery mechanics and fitters	Shop salesperson, non-food stores
4	Machine tool operators	Child-care workers
5	Home based personal care and related workers	Personal care and related workers not elsewhere classified
6	Lift truck operators	Nursing associate professionals not elsewhere classified
7	Railway brakers, signallers and shunters	Salesperson, stalls
8	Earth moving and related plant operators	Building caretakers
9	Helpers in restaurants	Protective services workers not else-where classified
10	Bus and tram drivers	Managers of small enterprises in wholesale and retail trade, hotels and restaurants, transport, communications
Med-low	Men	Women
1	Civil engineering technicians	Other office clerks
2	Physical engineering science technicians not elsewhere classified	Numerical clerks
3	Shop salesperson, non-food stores	Receptionists
4	Technical and commercial sales representatives	Pre-primary education teaching associate professionals
5	Power-production and related place operators	Technical and commercial sales representatives
6	Other office clerks	Office secretaries
7	Mechanical engineering	Recreation officers and related associate professionals
8	Finance and sales associate professionals not elsewhere classified	Finance and sales associate professionals not elsewhere classified
9	Managers of small enterprises not elsewhere classified	Physical engineering science technicians not elsewhere classified
10	Vocational teaching professionals	Government social benefits officials
Low	Men	Women
1	Supply distribution managers	Administrative secretaries and related associate professionals
2	Production and operations managers not elsewhere classified	Social workers and related associate professionals
3	Directors and chief executives	Production and operations managers not elsewhere classified
4	Specialist managers not elsewhere classified	Managers of small enterprises in public administration
5	Production and operations managers in manufacturing	Production and operations managers in public administration
6	Production and operations managers in education	Public service administrative professionals
7	Administrative secretaries and related associate professionals	Primary education teaching professionals

8	Production and operations managers in construction	Managers of small enterprises not elsewhere classified
9	Mechanical engineers	Production and operations managers in health and social work
10	Public service administrative professionals	Vocational teaching professionals
